# Mechanism-based management of taxane-induced neuropathic pain in breast cancer survivors: a critical review

**DOI:** 10.3389/fpain.2026.1717395

**Published:** 2026-04-13

**Authors:** Weydyson de Lima do Nascimento Anastácio, Tony Meireles dos Santos, Fabrício Oliveira Souto, Edmund O. Acevedo

**Affiliations:** 1 Centro de Ciências Médicas, Universidade Federal de Pernambuco, Recife, PE, Brazil; 2 Departamento de Educação Física, Universidade Federal de Pernambuco, Recife, PE, Brazil; 3 Instituto Keizo Asami, Universidade Federal de Pernambuco, Recife, PE, Brazil; 4 Department of Kinesiology and Health Sciences, Virginia Commonwealth University, Richmond, VA, United States

**Keywords:** breast cancer, chemotherapy-induced peripheral neuropathy, mechanism-based interventions, neuroinflammation, neuropathic pain, taxane

## Abstract

Taxane-induced neuropathic pain (TINP) is a debilitating condition and represents a significant therapeutic challenge in the treatment of breast cancer. The objective of this critical review is to initially explore the molecular and cellular mechanisms in the peripheral and central nervous systems associated with TINP, including mitochondrial dysfunction, oxidative stress, axonal degeneration, maladaptive neuroplasticity, and neuroimmune activation. The review then proposes to present clinical and experimental interventions that target these peripheral and central pathways, such as pharmacological treatments, invasive and non-invasive neuromodulation techniques, physical activity, manual therapies (including acupuncture and massage), and cryotherapy. Although clinical trials investigating these strategies have shown promising results, several methodological limitations must be considered, such as small sample sizes, heterogeneity in study designs, and the frequent classification of taxane-induced neuropathic pain as a secondary outcome. Therefore, it is concluded that there is a clear need for more methodologically rigorous studies, particularly those involving mechanism-oriented interventions, in the context of TINP rehabilitation in breast cancer.

## 1 Introduction

Breast cancer remains one of the most common malignancies affecting women. In the United States alone, an estimated 316,950 women were diagnosed with breast cancer, and 42,170 died from the disease in 2025 (1). Although advances in early detection and treatment have significantly improved survival rates, approximately 73% and 61% of survivors now live beyond five and ten years, respectively (2). Challenges remain, particularly in managing treatment related side effects.

Chemotherapy is a cornerstone in breast cancer treatment, employing cytotoxic agents to induce apoptosis and necrosis in cancer cells (3). Treatment modalities include neoadjuvant chemotherapy, administered prior to the primary intervention; adjuvant chemotherapy, delivered following initial treatment to reduce recurrence; and salvage chemotherapy, used when the disease is refractory or has recurred (3). Among the most effective chemotherapeutic agents are taxanes, such as paclitaxel, docetaxel (4), cabazitaxel and nanoparticle albumin-bound paclitaxel (nab-paclitaxel) (5). These agents have demonstrated efficacy in both early stage (6) and metastatic (7) breast cancers. Originally derived from the bark of the Pacific yew, a tree from the Taxaceae family native to North America, taxanes act by stabilizing microtubules, consequently, inhibiting normal cell cycle progression and inducing cell death (8). More recently, advances in production strategies have shifted taxane manufacturing from unsustainable plant extraction and limited semi-synthetic approaches to programmable biosynthesis in engineered cellular systems, enabling the scalable and sustainable development of novel derivatives (9).

However, the cytotoxic effects of taxanes are not exclusive to malignant cells. These drugs also affect healthy tissues, including the fibers of the somatosensory nervous system (10) leading to significant side effects such as taxane acute pain syndrome (TAPS) (11). TAPS occurs in the first two to three days after the start of chemotherapy (12, 13).

CIPN is a more clinically significant concern induced by neurotoxic agents and characterized by damage or dysfunction in the peripheral nerves (14). This condition can substantially impair patients' quality of life, often necessitating dose reductions, delays in treatment, or even premature discontinuation of therapy (15). Common symptoms include sensory, motor or autonomic deficiencies, with neuropathic pain as a central feature (16, 17). The primary sensory symptoms are symmetrical and often follow a distal 'stocking-and-glove' distribution, commonly beginning in the feet (18). A study involving 219 breast cancer survivors treated with paclitaxel reported that 97% experienced taxane-induced peripheral neuropathy, with persistence for up to three years in 41% of cases (19). Similarly, in a cohort of 127 survivors treated with docetaxel showed incidence rates of 31.5% for motor neuropathy and 21.3% for sensory neuropathy (20). In 188 survivors treated with the FEC-D regimen (Fluorouracil, Epirubicin, and Cyclophosphamide followed by docetaxel), moderate to severe pain occurred, primarily described as “throbbing”, but descriptors such as “burning”, “radiating”, and “sharp” were also used, with pain in cycle three predicting chronic pain (16). Together, these findings underscore the high incidence and severity of taxane-induced neuropathic pain (TINP).

**Figure 1:**
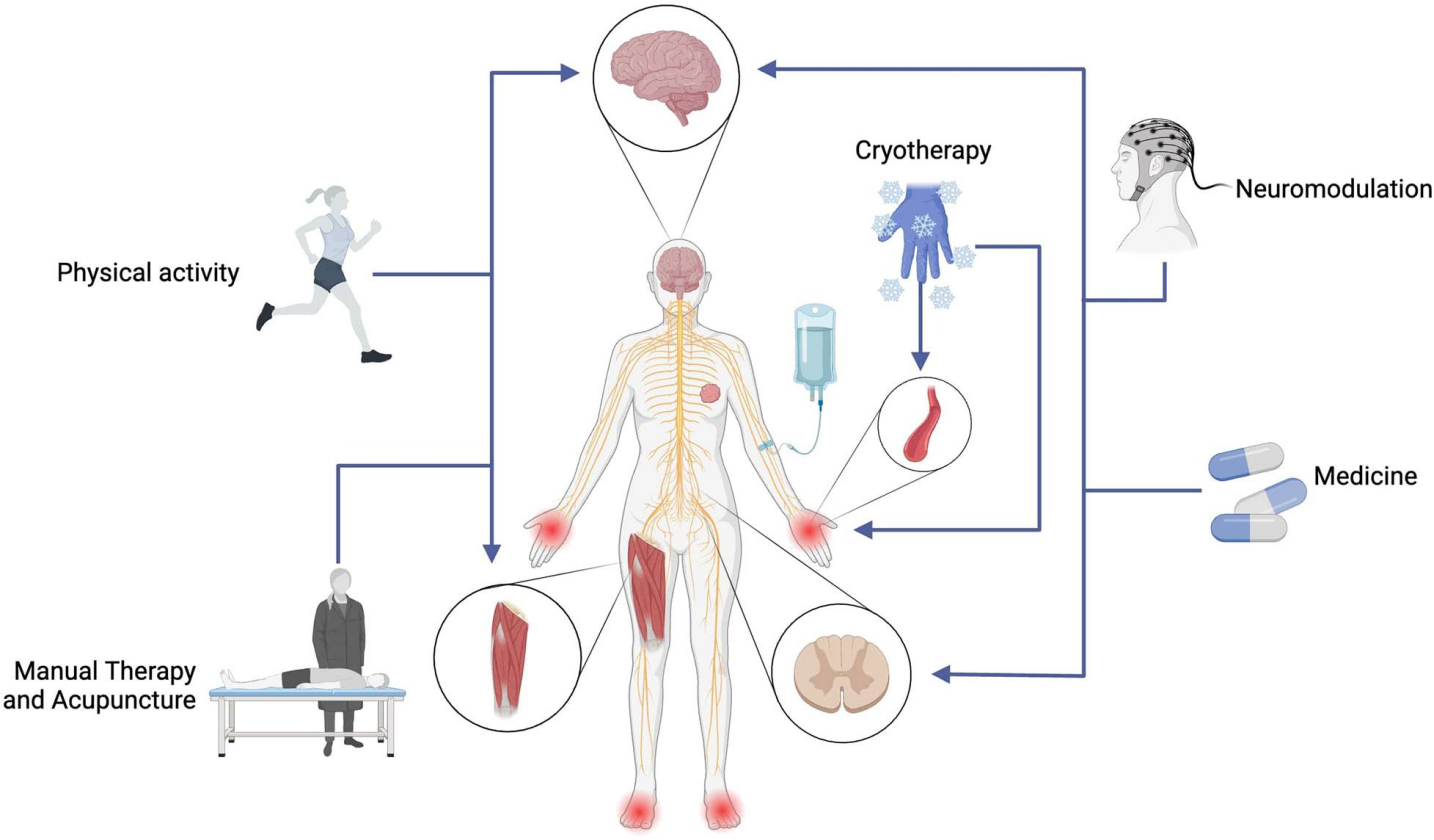
Interventions for the management of TINP. Taxane chemotherapy can induce neuropathic pain, significantly impacting the quality of life in breast cancer survivors. Alternative pain management strategies include medicine, physical activity and exercise, neuromodulation through invasive techniques such as vagus nerve stimulation (VNS), spinal cord stimulation (SCS), and intracranial self-stimulation (ICSS), as well as non-invasive electrical stimulation techniques like transcranial direct current stimulation (tDCS), transcranial magnetic stimulation (TMS), transcutaneous electrical nerve stimulation (TENS), and high-tone external muscle stimulation (HTEMS). Manual therapies such as massage and acupuncture, along with cryotherapy, are also employed. These approaches modulate neural activity and influence pain pathways, promoting analgesia. Further research is needed to clarify their mechanisms of action and optimize combined therapeutic strategies for chemotherapy-induced neuropathic pain.

Due to the pathophysiological complexity and challenging pharmacological management of TINP, exploring alternative strategies for its management becomes imperative. To address this unmet clinical need, a variety of pharmacological and non-pharmacological interventions have been investigated (as shown in Figure 1), including medications (21), neuromodulation techniques (22–30), physical activity and exercise (31), manual therapies (32), cryotherapy (23), and acupuncture (33). These approaches address different mechanisms of neuropathic pain, including inflammation, axonal degeneration, and central sensitization, showing variable effectiveness across populations while offering both complementary and standalone therapeutic potential.

However, there is a need for a greater understanding of how these interventions impact pain perception, the mechanistic processes, and the possibility that individual or combined use of these interventions may have beneficial effects in the TINP contexts. This paper aims to: (1) review the pathophysiological mechanisms underlying TINP and cell death; (2) evaluate current evidence-based interventions for managing chemotherapy-induced neuropathy considering this pathophysiology; and (3) identify limitations within the current literature and propose future directions for research to support the development of more effective and targeted therapeutic strategies.

## 2 Mechanisms of taxane-induced cell death

**Figure 2:**
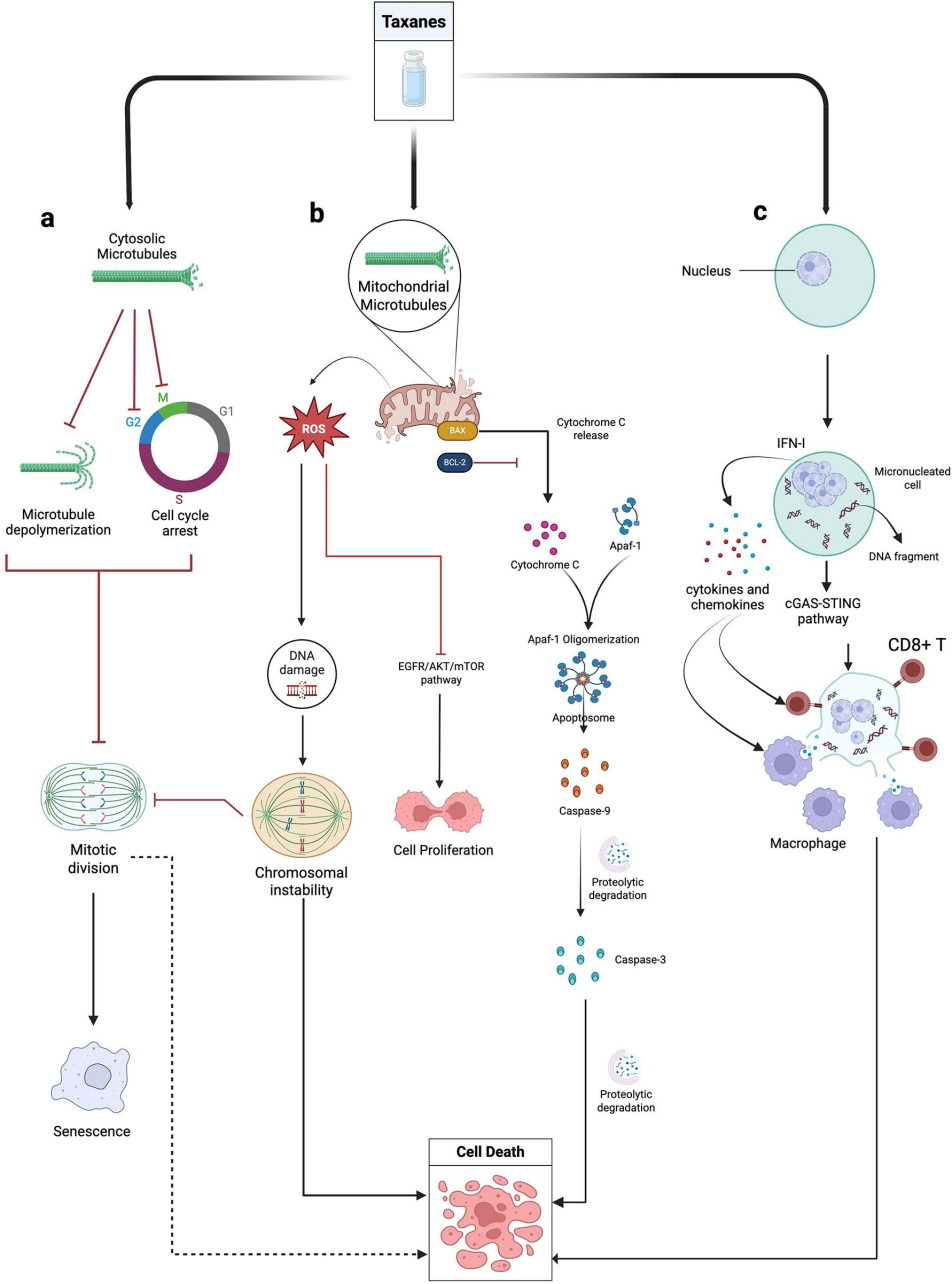
Mechanisms of taxane action in tumor cell death. Taxanes, such as paclitaxel and docetaxel, initiate the tumor cell death process through three main pathways: **(a)** stabilization of cytosolic microtubules, **(b)** stabilization of mitochondrial microtubules and **(c)** micronucleation. Taxanes stabilize microtubules in the cytosol and mitochondria, inducing apoptosis and cell cycle arrest. This mechanism triggers transient reactive oxygen species (ROS) generation, leading to DNA damage, chromosomal instability, and activation of the cGAS-STING pathway, that modulates immune responses. Taxane-induced micronucleation can result in nuclear membrane rupture, promoting cytokine production and tumor-specific CD8+ T cell activation. These effects contribute to both antitumor efficacy and the development of chronic neuropathic pain.

This section outlines the pathophysiology mechanisms underlying TINP, with a focus on cellular pathways that may inform therapeutic interventions. Taxanes are commonly administered intravenously every 1 to 3 weeks at doses from 175 to 225 mg/m² over 3 h for paclitaxel or between 75 and 100 mg/m² over 1 h for docetaxel (34). Although the full extent of their mechanisms of action is not yet completely understood, it is well established that taxanes primarily target microtubules (3), which are important cellular structures that regulate cell shape, locomotion, organelle transport, and chromosome segregation in mitosis (35). This initial mechanism of action can cause cell cycle arrest that prevents mitotic division. It is also believed that taxanes can induce intrinsic apoptosis leading to extensive proteolytic degradation and cell death (for additional description of the mechanism see Figure 2) (3).

A brief description of the mechanisms is that taxanes exert effects on the mitochondria within the cell inducing the transient generation of reactive oxygen species by inhibiting complex IV (cytochrome c oxidase) of the electron transport chain, as well as facilitating the opening of mitochondrial permeability transition pores by binding to mitochondrial tubulins, which allows the release of these reactive oxygen species (36). The reactive oxygen species released by mitochondria, induced by taxanes such as paclitaxel and docetaxel, can also cause DNA damage, leading to missegregation in proliferating cells, promoting chromosomal instability (37), and suppressing the pathway responsible for cell growth, proliferation, survival, and metabolism (EGFR/PI3 K/AKT/mTOR signaling pathway) (38).

Additionally, the stabilization of microtubules by taxanes can induce cell death through mechanisms related to micronucleation in cancer cells (3), a phenomenon in which chromosome fragments or whole chromosomes that are not incorporated into the main nuclei form multiple micronuclei in the cytoplasm (39). The formation of these multiple micronuclei can result in irreversible nuclear membrane ruptures, accumulation of DNA in the cytoplasm, and activation of the pathway responsible for gene stimulation [cGMP-AMP synthase—Stimulator of Interferon Genes (cGAS-STING) pathway] (40, 41). Consequently, this activation can lead to the production of type I interferon (IFN-I) which induces cytokine and chemokine production, modulating antigen presentation by tumor-associated macrophages and activating tumor-specific CD8+ T cells promoting an immunogenic environment (42). The cGAS-STING pathway prevents tumorigenesis by linking DNA damage to immune surveillance, senescence, and cell death (40).

As described, the effects of taxanes on microtubules and mitochondria trigger a cascade of cellular events that result in oxidative stress, genomic instability, and immune activation. Such mechanisms explain the antitumor efficacy of taxanes and help to understand their adverse effects. Thus, detailed knowledge of these pathways may guide therapeutic strategies to mitigate side effects, such as neuropathic pain, and optimize the clinical use of taxanes. Although the scope of the present study does not include an exhaustive review of the mechanisms by which taxanes induce cell death, elegant literature reviews describe these mechanisms in detail and can be consulted (3, 43).

## 3 Mechanisms and implications of TINP

**Figure 3:**
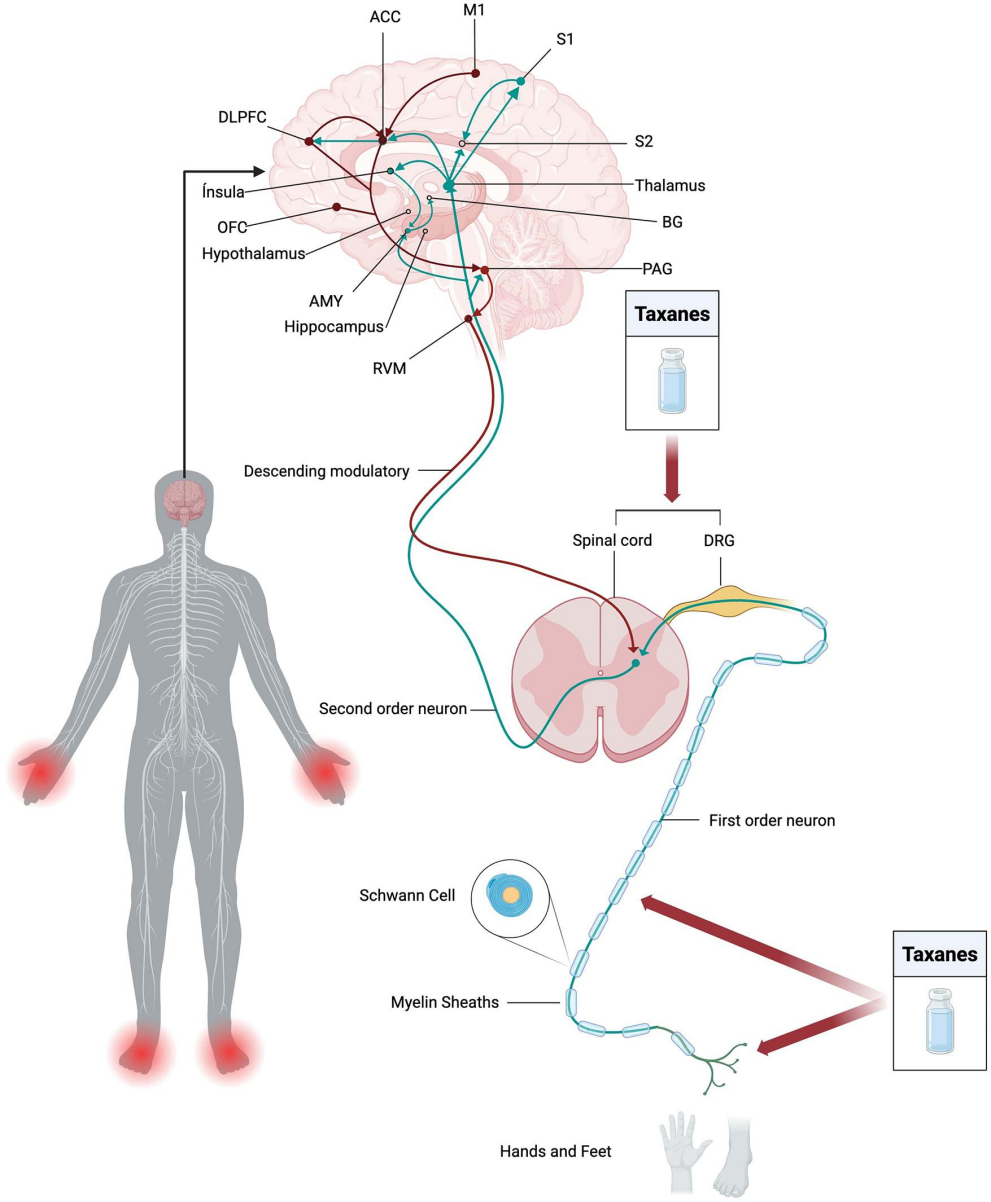
Mechanisms of TINP. Neuropathic pain arises from damage to the somatosensory system, affecting both the peripheral (PNS) and central nervous systems (CNS). Taxanes such as paclitaxel primarily target the PNS, damaging peripheral nociceptors and disrupting axonal transport. In the DRG and spinal cord, taxanes alter spinal synaptic transmission, generating maladaptive neuroplasticity in the CNS. These alterations compromises ascending pathways for nociceptive impulse conduction extending to cortical and subcortical regions, contributing to the chronicity of pain. This attack on pain-processing regions in the CNS further impairs descending pain control circuits, weakening inhibitory modulation. Thus, taxane-induced neuropathic pain results from a complex interplay of cellular, neuroinflammatory, and neuroplastic mechanisms. Neuroinflammation generated by the action of taxanes activates microglial cells, which release pro-inflammatory cytokines. These processes lead to neuronal sensitization, intensifying allodynia and hyperalgesia.

Neuropathic pain is characterized by damage or disease affecting the somatosensory system (44) which integrates the peripheral nervous system (PNS) and the central nervous system (CNS) to perceive touch, vibration, temperature, pain, and movement (45). Within this system, the perception of body position and movement is referred to as proprioception, whereas kinesthesia, one of its submodalities, is the conscious perception of joint movement (46). As presented above, taxane toxicity in somatosensory nerves can cause neuropathic pain (10) and it is believed that taxanes may induce neuronal apoptosis through the mechanisms similar to those present in cancer cells [e.g., microtubule stabilization, cytochrome C release, caspase activation, and calcium (Ca²^+^) dysregulation] (47). This section will describe the impact of taxanes on pain perception, starting with the damage to nociceptors; followed by disruptions in signal transduction, conduction, and spinal transmission; and maladaptive neuroplastic changes in the central nervous system, leading to chronic neuropathic pain. Figure 3 illustrates the components discussed below.

Pain signaling occurs in four steps: transduction, transmission, modulation, and perception (48). It begins with transduction, where peripheral nociceptors detect harmful stimuli and release substances such as glutamate and pro-inflammatory peptides like substance P, and calcitonin gene-related peptide (CGRP) (49). These mediators promote vasodilation, immune activation, and sensitization of nociceptors (50–52). As presented above, taxanes contribute to this process by inducing metabolic changes such as oxidative stress and mitochondrial dysfunction in peripheral nerve endings (53). In cultured cells treated with paclitaxel, mitochondrial morphological abnormalities have been observed, suggesting vacuolar degeneration (54).

Following transduction, conduction occurs as nociceptive signals travel through myelinated A*δ* and unmyelinated C fibers toward the spinal cord (55). Data derived from *in vitro* models suggest that taxanes disrupt this process by impairing axonal transport and growth, reducing axonal length by approximately 50% and altering neuronal morphology (54). Additionally, paclitaxel sensitizes A*β* fibers (active at the beginning and end of a continuous mechanical stimulus) (56) and A*δ*, contributing to mechanical allodynia (a non-painful stimulus producing a sharp pain response) and hyperalgesia (increased perception of pain) (57, 58).

Structural alterations in axons and myelin sheaths may also occur (47). Schwann cells play a crucial role in this process by producing the myelin sheath, which facilitates nerve conduction and increases the speed of electrical impulse propagation in axons (59). When neurons are injured, activated Schwann cells change their phenotype and stimulate axonal growth and regeneration (60). Moreover, these cells modulate neuropathic pain through receptor expression and the release of molecules (e.g., growth factors, cytokines, and ATP), and demyelination (61). Interestingly, Imai, Koyanagi (62) showed that paclitaxel affected cultured rat Schwann cells in a concentration-dependent manner, with 0.01 μM reducing the number of Myelin Basic Protein (MBP)-positive cells, responsible for forming and maintaining the myelin sheath in the peripheral nervous system. The same concentration also decreased neuronal density, reduced dendritic complexity, and shortened neuritic processes. paclitaxel also induced morphological alterations in Schwann cells, including bipolar process retraction and a rounded cell shape. Additionally, both paclitaxel and docetaxel promoted Schwann cell dedifferentiation, evidenced by increased expression of p75 neurotrophin receptor (p75NTR) and galectin-3, markers of immature and dedifferentiated phenotypes, respectively.

As the signal reaches the dorsal horn of the spinal cord, transmission takes place through synaptic integration. The first-order neurons synapse with second-order neurons at the dorsal horn of the spinal cord (63), modulated by neurotransmitters [e.g., substance P, glutamate, calcitonin gene-related peptide (CGRP), and ATP], which activate postsynaptic receptors [N-methyl-D-aspartate [NMDA] and *α*-amino-3-hydroxy-5-methyl-4-isoxazolepropionic acid [AMPA] receptors] (49).

Table 1 outlines the primary mechanisms within the peripheral nervous system associated with TINP. The following pathways and mechanisms have been investigated in relation to the perception of TINP at this stage:

(a) Paclitaxel alters pain transmission by increasing the release of glutamate, related to NMDA and non-NMDAR (AMPA and kainate) receptors, serotonin (5-HT), substance P, CGRP, and activates adrenergic and opioid receptors, intensifying neuropathic pain (64).
(b) Paclitaxel exacerbates pain transmission by increasing the expression of ion channels such as Transient Receptor Potential Vanilloid 1 (TRPV1) in the spinal cord (65), and TRPV4 and Transient Receptor Potential Ankyrin 1 (TRPA1) in the dorsal root ganglion (DRG) (66), heightening neuronal excitability.
(c) Paclitaxel increases Ca*α*2*δ*-1 (Ca2 + receptor) expression in medium/large-diameter DRG neurons, regulating Ca²^+^ influx and activating sensory neurons (57). Nerve damage promotes Ca*α*2*δ*-1 binding to thrombospondin-4 (TSP4) or NMDAR, increasing Ca²^+^ influx, activating the protein kinase C, and modulating TRPA1 and TRPV1 channels, implicated in neuropathic pain (67).
(d) Paclitaxel upregulates ATF3 (activating transcription factor 3), a nerve injury marker (68), and caspase-3, a regulator of neuronal death and synapse pruning (69), predominantly in large lumbar DRG neurons compared to thoracic DRG neurons in rats; however, it appears that nerve damage is dependent on higher doses of paclitaxel (64).

**Table 1:** Mechanisms of TINP in the peripheral nervous system.

Structure/Location	Molecular Mechanisms and Cellular Alterations
Peripheral Terminals	Oxidative stress; mitochondrial dysfunction with vacuolar degeneration; excessive release of Substance P, CGRP, and glutamate.
Peripheral Nerves	Reduction in axonal length; morphological changes in the myelin sheath; sensitization of myelinated A*δ* and A*β* and unmyelinated (C) fibers; reduction in MBP + Schwann cells and cellular dedifferentiation (increased p75-IR and galectin-3).
Neuronal Soma (DRG)	Upregulation of TRPA1 and TRPV4 channels; increased expression of the calcium channel *α*2δ1 subunit; expression of injury and apoptotic markers (ATF3, caspase-3); activation of necroptosis via RIP3/MLKL signaling.

ATF3, activating transcription factor 3; CGRP, calcitonin gene-related peptide; DRG, dorsal root ganglion; MBP, myelin basic protein; MLKL, mixed lineage kinase domain-like pseudokinase; p75-IR, p75 immunoreactivity; RIP3, receptor-interacting protein kinase 3; TRPA1, transient receptor potential ankyrin 1; TRPV4, transient receptor potential vanilloid 4.

Then at the spinothalamic tract, ascending pathways are responsible for nociceptive signaling. Afferent nociceptive input reaches the brain via spinal pathways and projections from the thalamus to the insula, anterior cingulate cortex (ACC), primary somatosensory cortex (S1), and secondary somatosensory cortex (S2), and from the amygdala to the basal ganglia (70). The medial spinothalamic tract conducts motivational and affective signals, including the cognitive, emotional and autonomic aspects, to the thalamic nuclei, periaqueductal gray matter (PAG), somatosensory cortex and limbic centers. Whereas the lateral spinothalamic tract conducts sensory discriminative aspects, including intensity, localization and character of pain to the ventral posterolateral thalamic nucleus, ventral posterior inferior nucleus and the postcentral gyrus (71). The medial pathway processes the emotional component of suffering, involving the rostral and dorsal anterior cingulate cortex (rdACC) and the anterior insular cortex, whereas the lateral pathway is associated with sensory perception and connects to the somatosensory cortex (71). Although taxanes have limited ability to cross the blood-brain barrier (72, 73), patients also present with symptoms of taxane-induced central neurotoxicity affecting both the spinal cord and higher brain centers (10).

At the neural level, pain involves interconnected circuits that integrate sensory, emotional, and cognitive components. Chronic pain, including neuropathic pain, leads to functional and morphological brain reorganization, affecting specific regions like the mesocorticolimbic circuitry and hippocampus. While traditionally linked to memory and learning, the hippocampus also integrates cognition and emotion, with its functional connectivity reorganized in chronic pain, influencing sensory and affective processing. The connectivity between the nucleus accumbens and medial prefrontal cortex can predict the transition to chronic pain, and the hippocampus appears to be linked to pain exacerbation (74).

As an example, in type 1 diabetic rats with neuropathic pain, upregulation of hippocampal purinergic receptor P2X4 (P2X4R) has been shown to activate microglia, increasing neuroinflammation and causing neuronal damage and hyperalgesia (75). Furthermore, chronic pain deactivates the dentate gyrus in the dorsal hippocampus, which normally regulates both sensory (tactile allodynia) and emotional (motivational/affective) aspects of pain (76). Notably, the hippocampus may be susceptible to paclitaxel neurotoxicity (10) which suggests its involvement in neuropathic pain caused by taxanes.

The processing of pain is not purely sensory but is significantly influenced by higher-order cognitive and affective domains. Emotional states such as anxiety and fear, along with maladaptive cognitions like catastrophizing, modulate central pain pathways and contribute to both pain chronification and poorer treatment outcomes (77, 78). The ACC and lateral prefrontal cortex contribute to emotional and cognitive control, while the anterior insula is activated in reappraisal strategies, and the thalamus and primary somatosensory cortex are essential for sensory processing. Furthermore, the amygdala integrates emotional and sensory information, influencing affective responses (74).

Patients with taxane-induced peripheral neuropathy may exhibit pre-existing alterations in brainstem activity, predisposing them to chronic pain development (79). For example, functional imaging studies have revealed hyperactivity in the PAG, thalamus, ACC, and somatosensory cortex in individuals experiencing taxane-induced peripheral neuropathy (80). In addition, Nashawi, Masocha (81) demonstrated that paclitaxel induced mechanical allodynia and increased synaptic excitability in the ACC. These effects were associated with dysfunction in GABAergic neurotransmission, particularly involving GABAb receptors, suggesting a role in the supraspinal modulation of synaptic transmission. Similarly, Masocha (82) showed that paclitaxel significantly increased the expression of sodium channel alpha (Nav1.1, Nav1.2, Nav1.6, and Nax) and beta (Nav*β*4, Nav*β*1, and Nav*β*3) subunits in the ACC of mice. The most highly upregulated subunits were Nav1.2 and Nax among the alpha subunits, and Nav*β*3 among the beta subunits. Taken together, these findings suggest that both GABAergic dysfunction and increased sodium channel expression contribute to ACC hyperexcitability during neuropathic pain.

Regarding the emotional component of pain, Liu, Li (83) showed that paclitaxel induced neuropathic pain and anxious behaviors in mice, accompanied by a significant increase in c-Fos-positive neurons in the basolateral and central amygdala (indicating neuronal activation in these regions) is associated with chronic pain. The central amygdala receives projections from glutamatergic neurons expressing calcium/calmodulin-dependent protein kinase II (CaMKII) originating in the basolateral amygdala. Selective inhibition of CaMKII-positive neurons in these regions has demonstrated reduced hyperalgesia and anxious behaviors, indicating their influence on pain and negative emotions.

Thus, chronic pain results from the dynamic interaction of these circuits, highlighting its multidimensional nature and the need to understand its mechanisms to develop more effective therapeutic approaches. The multidimensional nature of chronic pain has led to propose the Triple Network Model in pain, which includes the Default Mode Network, Salience Network, and Central Executive Network. The Default Mode Network integrates pain into self-identity, the Salience Network amplifies emotional responses to pain, and the Central Executive Network supports cognitive control, which can be impaired by chronic pain. Together, these networks explain the complex relationship between the sensory, emotional, and cognitive dimensions of chronic pain (48). The main mechanisms within the spinal cord and CNS implicated in TINP are summarized in Table 2.

**Table 2:** Mechanisms of TINP in the spinal cord and central nervous system.

Structure/Location	Molecular Mechanisms and Cellular Alterations
Spinal Cord	Increased release of excitatory neurotransmitters in the dorsal horn; activation of NMDA and AMPA receptors; impaired descending modulation via adrenergic and opioid receptors; neuroinflammation driven by microglial activation.
Spinothalamic Tract	Divergent signaling transmission: Lateral Pathway (sensory-discriminative) and Medial Pathway (affective-emotional) involving thalamic nuclei.
Supraspinal Regions	ACC: GABAergic neurotransmission dysfunction and upregulation of voltage-gated sodium channels (Nav 1.1, 1.2, 1.6) and Nax.
	Amygdala: Neuronal activation (c-Fos expression) and increased CaMKII associated with anxiety-like behaviors.
Functional Connectivity	Disruption of the Triple Network Model: altered dynamic interactions between the Default Mode Network, Salience Network, and Central Executive Network.

ACC, anterior cingulate cortex; AMPA, alpha-amino-3-hydroxy-5-methyl-4-isoxazolepropionic acid; ATP, adenosine triphosphate; CaMKII, Ca2 + calmodulin-dependent protein kinase II; NMDA, N-methyl-D-aspartate; Nav, voltage-gated sodium channels; Nax, sodium-sensing channel; P2X4R, purinergic receptor P2X 4.

Beyond the peripheral nervous system, neuropathic pain has a strong neuroimmune component, with inflammatory mediators such as interleukin (IL)-1β, IL-6, and tumor necrosis factor (TNF)-*α* contributing to pain hypersensitivity (84). Taxanes likely activate the inflammatory cascade, causing peripheral nerve damage and altering pain sensitivity, but categorizing pain as exclusively inflammatory or neuropathic oversimplifies this process (16). While studying this process in humans remains challenging because of cancer and chemotherapy, rodent models suggest that immune and glial cell activation play a key role in TINP (60). The neuroimmune inflammatory pathways have also been investigated in the literature. Below is a brief explanation of several neuroinflammatory pathways associated with the neuroimmune component of TINP:

(a) Paclitaxel-treated rats have exhibited increased toll-like receptor (TLR) 4 expression in L4-5 CGRP- and IB4-positive DRG small neurons and the spinal cord, with myeloid-differentiation response gene 88 (MyD88) upregulated only in CGRP-positive neurons and toll/interleukin 1 receptor domain-containing adapter-inducing interferon-*β* (TRIF) in both CGRP- and isolectin B4-positive neurons, alongside mechanical hypersensitivity (85).
(b) Paclitaxel has been shown to drive macrophage accumulation in the DRG via metalloproteinase-3 (MMP-3) and chemotactic factors, while CD8+ T cells and IL-10 aid allodynia recovery (64). In this sense, Ma, Wang (86) showed that paclitaxel also increases CD68 + macrophage infiltration in the DRG, elevating TNF-α and IL-1β levels, while also upregulating the mRNA expression of receptor-interacting protein kinase 3 (RIP3), mixed-lineage kinase domain-like (MLKL) protein, and phosphorylated pseudokinase mixed lineage kinase domain-like (p-MLKL) proteins, promoting cell necrosis in the DRG of rats. Ma, Zhao (87) also showed that paclitaxel induces necroptosis in DRG neurons in a RIP3/MLKL-dependent manner in rats, which is associated with increases in TNF-α and IL-1β following paclitaxel injection, a process that was successfully blocked by necrostatin-1 (Nec-1).
(c) High mobility group box 1 (HMGB1) is a protein that regulates chromosomal structure in the nucleus, sustains autophagy in the cytoplasm, and mediates inflammation and immunity extracellularly, functioning as a damage-associated molecular pattern molecules (DAMP) (88). paclitaxel-induced neuropathic pain also is mediated by HMGB1 release from macrophages (89). In this context, paclitaxel induced cytoplasmic translocation and HMGB1 release via oxidative stress and activation of p38MAPK/NF-*κ*B/HAT (89) represent a stress-activated pathway (90). Macrophage-derived HMGB1 can activate some immune receptors [receptor for advanced glycation endproducts [RAGE], C-X-C chemokine receptor type 4 [CXCR4], and TLR4] and neuronal humoral factors amplify this release (89).
(d) Other kinds of TLR receptors can be involved in TINP. For example, TLR9 is a receptor for CpG motifs involved in immune response autoantibody production (91). TLR9 signaling in macrophages regulates chemotherapy-induced mechanical allodynia in male mice through neuron-macrophage interactions, and paclitaxel induced mechanical allodynia via TLR9, independent of TLR4, leading to macrophage infiltration in DRG and increased TNF and CXCL1 in a sex-dimorphic manner (92). This suggests that the CXCL1/CXCR2 pathway may contribute to neuropathy, while CIPN in females could depend on TLR9 in an immunodeficient context.

As shown above, the neuroimmune interaction in the context of taxane-induced peripheral neuropathy and, consequently, neuropathic pain is complex. Excellent reviews of the literature help to explain in more detail the processes related to their interaction (60, 64, 93). Table 3 shows the main components of neuroimmune activation.

**Table 3:** Mechanisms of neuroimmune activation.

Category/Pathway	Structure/Location	Key Mediators and Effects
Macrophage Activation	DRG and Peripheral Tissues	Infiltration of CD68 + macrophages mediated by MMP-3, CCL2, and CX3CL1 signaling; activation of TLR4 and TLR9 pathways.
cGAS-STING Pathway	Systemic/Various Tissues	Recognition of cytosolic DNA (from mitochondrial leakage) triggering the production of Type I interferons and pro-inflammatory cytokines.
Cytokines and Chemokines	PNS-CNS Axis	Elevated levels of TNF-α, IL-1β, and HMGB1; TLR9-induced allodynia.
Glial and T-cell Modulation	Spinal Cord	Microglial activation leading to BDNF release (signaling via p75 and TrkB receptors); role of CD8+ T-cells and IL-10 in the resolution of neuropathic pain.

BDNF, brain-derived neurotrophic factor; CCL2, CC-chemokine ligand 2; cGAS-STING, cyclic guanosine monophosphate-adenosine monophosphate synthase-stimulator of interferon genes; CX3CL1, C-X3-C motif chemokine ligand 1; DRG, dorsal root ganglion; HMGB1, high mobility group box 1; IL, interleukin; MMP-3, matrix metalloproteinase-3; p75, p75 neurotrophin receptor; TLR, Toll-like receptor; TNF-α, tumor necrosis factor-alpha; TrkB, tropomyosin receptor kinase B.

Further contributing to pain chronification, paclitaxel induces microglial dysregulation (94) and overexpression of the P2X4R, both involved in pain and neuroinflammation (95). This dysregulation and overexpression, leads to BDNF overexpression, sensitization of dorsal horn neurons and mechanical allodynia (94). BDNF modulates neuropathic pain via p75 and TrkB receptors, that enhance the release of glutamate and GABA, activating Ca²^+^ channels, and interacting with NMDA, AMPA, and GABA receptors. BDNF elevation in the spinal dorsal horn, along with microglia activation, drives hyperalgesia and central sensitization; however, its causal role in chronic pain in humans remains unconfirmed (96). This seems to indicate that maladaptive changes may occur in the central nervous system as a complex extension of the damage generated in the periphery in the process of pain chronification (97).

These maladaptive changes enhance central sensitization, disrupting the descending pain control pathway, responsible for inhibitory control of pain by modulating nociceptive signal transmission (71, 98). Descending systems are modulated by psychological factors via inputs from the ACC, prefrontal cortex (PFC), and amygdala that converge on the PAG (70). Normally, the PAG modulates nociceptive transmission through serotonergic and noradrenergic circuits, which inhibit pain signals at the spinal level (71, 98). These circuits modulate pain based on emotional and cognitive factors, activating the opioid system with serotonin and noradrenaline, while opioid receptors reduce neurotransmission and cell excitability (98). However, taxanes impair these circuits, weakening the inhibitory control of pain (99). Neuroplastic alterations in chronic pain states result in strengthened excitatory pathways and diminished inhibitory pathways, exacerbating pain persistence (100).

As presented in this session, TINP pain results from a complex interplay of peripheral and central mechanism. Peripherally, taxanes cause oxidative stress and mitochondrial dysfunction in nerve endings, impair axonal transport, reduce axonal length, and sensitize nerve fibers such as A*δ* and A*β*. They also induce structural changes and dedifferentiation in Schwann cells, which affect myelination and regeneration. In the DRG, there is increased expression of ion channels such as TRPA1, TRPV4, and Ca*α*2*δ*-1, upregulation of ATF3 and caspase-3, RIP3/MLKL-mediated necroptosis, accumulation of macrophages via MMP-3 and chemotactic factors, with elevation of inflammatory cytokines such as TNF-α and IL-1β. In addition, there is release of HMGB1 and activation of TLR4 and TLR9. Centrally, there is increased release of excitatory neurotransmitters (glutamate, substance P, CGRP, ATP) and microglial activation with overexpression of BDNF in the spinal cord, resulting in central sensitization. In higher brain regions, functional and morphological reorganization occurs, with hyperactivity (e.g., ACC, thalamus, and PAG) and increased synaptic excitability (e.g., GABAergic dysfunction, overexpression of sodium channel subunits in the ACC). Complementarily, neuroinflammation and dysregulation of descending pain control pathways contribute to pain chronicity and persistence, evidencing a complex cellular, neuroimmune, and modulatory interplay. Addressing these mechanisms requires a multidisciplinary approach that includes neuroprotective strategies, targeted therapies that address different mechanisms that explain chronic pain perception, and an improved understanding of individual susceptibility to TINP.

## 4 Management of taxane-induced neuropathic pain

The interventions identified and described below aim to address peripheral, central, and systemic mechanisms involved in the management of TINP. A literature search was conducted in the PubMed database to identify clinical studies related to each intervention independently. A consistent search strategy was applied across all interventions. Searches combined general terms (for breast cancer, taxanes, and neuropathic pain), combined with specific terms for each type of intervention (medications, neuromodulation, physical activity and exercise, neuromodulation, manual therapies, cryotherapy, and acupuncture). Studies including TAPS were not explicitly excluded from the literature search when pain outcomes were reported or could not be clearly distinguished from TINP. The selected publications were limited to clinical studies that analyzed TINP as a primary or secondary outcome, published in English in the last 10 years and filtered by the presence of the search terms in “title/abstract”. The search terms used are presented in Table 4. All studies described below are detailed in Supplementary Tables S1A–S1E.

**Table 4:** Research terms.

Category	Research Terms
Breast cancer	(((((((((((((((“Breast Neoplasm"[Title/Abstract]) OR (“Breast Tumors"[Title/Abstract])) OR (“Breast Tumor"[Title/Abstract])) OR (“Breast Cancer"[Title/Abstract])) OR (“Mammary Cancer"[Title/Abstract])) OR (“Mammary Cancers"[Title/Abstract])) OR (“Cancer of Breast"[Title/Abstract])) OR (“Cancer of the Breast"[Title/Abstract])) OR (“Cancer of the Breast"[Title/Abstract])) OR (“Human Mammary Carcinomas"[Title/Abstract])) OR (“Human Mammary Neoplasms"[Title/Abstract])) OR (“Breast Carcinoma"[Title/Abstract])) OR (“Breast Carcinomas"[Title/Abstract]))
Taxanes	(((((Taxane*[Title/Abstract]) OR (Docetaxel[Title/Abstract])) OR (Paclitaxel[Title/Abstract])) OR (Cabazitaxel[Title/Abstract])) OR (“Nab paclitaxel"[Title/Abstract])) OR (“Nab-paclitaxel"[Title/Abstract])
Taxane-induced neuropathic pain	(((((((((((“neuropathic pain"[Title/Abstract]) OR (“peripheral neuropathy"[Title/Abstract])) OR (“chemotherapy-induced neuropathic pain"[Title/Abstract])) OR (“chemotherapy-induced neuropathy"[Title/Abstract])) OR (“taxane-induced peripheral neuropathy"[Title/Abstract])) OR (“chemotherapy-induced peripheral neuropathy"[Title/Abstract])) OR (“taxane-induced peripheral neuropathy"[Title/Abstract])) OR (“docetaxel-induced peripheral neuropathy"[Title/Abstract])) OR (“paclitaxel-induced peripheral neuropathy"[Title/Abstract])) OR (“docetaxel-induced neuropathic pain"[Title/Abstract])) OR (“paclitaxel-induced neuropathic pain"[Title/Abstract])))
Medications	(((((((((((“medication*"[Title/Abstract]) OR (“medicine"[Title/Abstract])) OR (“pharmacotherapy"[Title/Abstract])) OR (“drug*"[Title/Abstract])) OR (“anticonvulsant"[Title/Abstract])) OR (“lidoderm"[Title/Abstract])) OR (“lyrica"[Title/Abstract])) OR (“cannabinoids"[Title/Abstract])) OR (“opioids"[Title/Abstract])) OR (“serotonin reuptake inhibitor"[Title/Abstract])) OR (“analgesic"[Title/Abstract])) OR (“topical agent"[Title/Abstract])
Neuromodulation	(((((((((((((((Neuromodulation*[Title/Abstract]) OR (“Electric Stimulation Therapy"[Title/Abstract])) OR (“Electric Stimulation"[Title/Abstract])) OR (“Transcranial Magnetic Stimulation"[Title/Abstract])) OR (“Repetitive Transcranial Magnetic Stimulation"[Title/Abstract])) OR (rTMS[Title/Abstract])) OR (“Transcranial Direct Current Stimulation"[Title/Abstract])) OR (tDCS[Title/Abstract])) OR (“Deep Brain Stimulation"[Title/Abstract])) OR (“Vagus Nerve Stimulation"[Title/Abstract])) OR (“Spinal Cord Stimulation"[Title/Abstract])) OR (“Cranial Electrotherapy Stimulation"[Title/Abstract])) OR (“Brain Stimulation"[Title/Abstract])) OR (Electrotherapy[Title/Abstract]))))
Physical activity and exercise	(((((((Exercise[Title/Abstract]) OR (Physical Activity[Title/Abstract])) OR (Physical Activities[Title/Abstract])) OR (Physical Exercise[Title/Abstract])) OR (Physical Exercises[Title/Abstract])) OR (Exercise Training[Title/Abstract])) OR (Exercise Trainings[Title/Abstract]))
Manual Therapy	((((((((((((Massage[Title/Abstract]) OR (Manipulations[Title/Abstract])) OR (“Craniosacral Massage"[Title/Abstract])) OR (Reflexology[Title/Abstract])) OR (“Manipulation Therapy"[Title/Abstract])) OR (“Manipulative therapies"[Title/Abstract])) OR (“Manipulative therapy"[Title/Abstract])) OR (“manual therapies"[Title/Abstract])) OR (“Manual Therapy"[Title/Abstract])) OR (Bodywork[Title/Abstract])) OR (Bodyworks[Title/Abstract])) OR (Rolfing[Title/Abstract]))
Cryotherapy	(((((Cryotherapy*[Title/Abstract]) OR (“Cold therapy"[Title/Abstract])) OR (Cryostimulation[Title/Abstract])) OR (“Whole-body cryotherapy"[Title/Abstract])) OR (“Partial-body cryotherapy"[Title/Abstract]))
Acupuncture	((((((((((“Acupuncture Treatment"[Title/Abstract]) OR (“Acupuncture Treatments"[Title/Abstract])) OR (Pharmacoacupuncture[Title/Abstract])) OR (“Pharmacoacupuncture Therapy"[Title/Abstract])) OR (Acupotomy[Title/Abstract])) OR (Acupotomies[Title/Abstract])) OR (Electroacupuncture[Title/Abstract])) OR (“Trigger Point*"[Title/Abstract])) OR (“Dry Needling*"[Title/Abstract])) OR (“Needling*"[Title/Abstract]))

### 4.1 Medications

About one-third of cancer survivors do not receive analgesia proportional to their pain intensity (101). In the specific management of taxane-induced neuropathy, agents established for other etiologies, such as amitriptyline and gabapentin, demonstrate limited efficacy (102). This gap in evidence in support of specific medication(s) is reflected in clinical practice. A large-scale observational study involving 1,516 breast cancer survivors treated with taxanes found that gabapentin (74.4%) and pregabalin (22.3%) were the most frequently prescribed medications. Notably, many breast cancer survivors (67.6%) received antineuropathic agents as monotherapy. Treatment durations were highly variable, with an average of 49 days. Notably, 39.2% of patients used the medication for less than one month, while 4.8% continued treatment for over a year (21). However, although pregabalin has shown some potential (102), it is not currently formally recommended by clinical guidelines for the treatment of taxane-induced neuropathy.

In direct contrast to the limited empirical evidence supporting the use of certain agents, duloxetine stands out as the only pharmacological treatment formally recommended for taxane-induced peripheral neuropathy, as endorsed by the American Society of Clinical Oncology (ASCO) (103). This recommendation is strongly supported by the results of a phase III randomized clinical trial (RCT), which demonstrated that duloxetine significantly reduced neuropathic pain in patients with platinum- or TINP (104).

Clinical studies have explored the use of pharmacological interventions to prevent or manage taxane-induced peripheral neuropathy, with particular attention to their impact on TINP in breast cancer survivors. We conducted a PubMed search that identified three studies published within the past ten years addressing both preventive and therapeutic interventions for neuropathy, in which neuropathic pain was evaluated as a primary or secondary outcome.

Holotiuk, Kryzhanivska (105) conducted a RCT to investigate the efficacy of the combination of alpha-lipoic acid (ALA) and ipidacrine hydrochloride in preventing taxane-induced peripheral neuropathy (by paclitaxel) in 32 breast cancer survivors in stages II-III. The survivors underwent six cycles of neoadjuvant chemotherapy with paclitaxel, doxorubicin, or epirubicin every three weeks. The intervention consisted of administering ALA capsules (600 mg) once daily in the morning, before meals, and ipidacrine hydrochloride (20 mg) three times daily during chemotherapy cycles, with interruption two days before and four days after chemotherapy. The results showed a reduction in the severity of taxane-induced peripheral neuropathy, particularly in sensory symptoms such as numbness, paresthesia, and TINP. The medication group showed improvement in sensory symptoms, including TINP, of 15.75% (mean severity: ALA = 1.12 vs. control = 1.75; *p* < 0.01) and 25% (ALA = 1.62 vs. control = 2.62; *p* < 0.01) after the third and sixth chemotherapy cycles, respectively. Interestingly, a significant correlation was observed between the reduction in deep tendon reflexes and the severity of sensory symptoms [control group: after cycle 3: rx,y = 0.83 ± 0.15; after cycle 6: rx,y = 0.78 ± 0.17; medication group: after cycle 6: rx,y = 0.83 ± 0.15 (*p* < 0.05)]. The results did not show significant improvements in motor symptoms, muscle contraction, or in the reduction of the compound action potential amplitude in the peroneal nerve (for all, *p* > 0.05). Methodologically, while this RCT demonstrated prophylactic benefits for sensory symptoms, its small sample size (*N* = 32) and lack of clear double-blinding, introduce significant risks of observer and treatment-related biases. These limitations constrain the reliability of the findings and underscore the need for larger, multi-center trials to validate the efficacy of alpha-lipoic acid and ipidacrine  .

Another RCT conducted by Aghili, Taherioun (106) investigated the preventive effects of duloxetine on taxane-induced peripheral neuropathy and its implications for dose adjustment or treatment discontinuation. Forty-seven breast cancer patients participated in the study, who underwent chemotherapy with paclitaxel (175 mg/m^2^). The intervention consisted of administering 30 mg/day of duloxetine in the first week, escalating to 60 mg in two doses in the second week of each cycle (1 h before paclitaxel, continuing for 14 days). The control group followed the same protocol and received placebo. It was observed that more patients reported neuropathy in the control group than in the duloxetine group (*N* = 8 vs. *N* = 16; chi-squared = 4.78, *p* = 0.029), with lower severity (*F* = 8.01, *p* = 0.007) and lower occurrence of new neuropathies [duloxetine group [N] = 8 vs. control group [N] = 16] in the duloxetine group, supporting its efficacy. Furthermore, duloxetine demonstrated significantly superior pain control at different time points in the study compared to placebo (*p* = 0.027), although both groups exhibited improvement in pain over time (duloxetine: *F* = 18.35, *p* < 0.001; control: *F* = 21.16, *p* < 0.001). Sensory symptoms such as numbness (*F* = 0.56, *p* = 0.457) or paresthesia (*F* = 0.63, *p* = 0.43) did not show differences between the groups. The results suggest that duloxetine is effective in preventing taxane-induced peripheral neuropathy, and to promote a more effective control of TINP. Furthermore, duloxetine demonstrated more pronounced effects on objective measures, such as nerve conduction. It is worth noting that the sample size was modest for a Phase II clinical trial (*N* = 47), which limited the generalizability of the results.

Mirogabalin was another medication tested in a clinical trial. Misawa, Denda (107) evaluated the efficacy of mirogabalin in treating pain in patients who developed oxaliplatin- or taxane-induced peripheral neuropathy across four types of cancer, including breast (Breast cancer [11.5%]; colorectal cancer [65.4%]; non-small-cell lung [13.5%]; gastric cancer [9.6%]). Oxaliplatin- or TINP was analyzed as a secondary outcome. Fifty-two survivors (42% women) with grade ≥ 2 neuropathy according to the Common Terminology Criteria for Adverse Events version 5 (CTCAE v.5) participated in the study. Mirogabalin was administered for 12 weeks, starting at an initial dose of 5 mg twice daily, which was then titrated based on tolerance and renal function. The results showed that despite the overall reduction in pain over time, the effects for taxanes were limited. The mean pain score by the Numeric Rating Scale (NRS) dropped from 5.5 ± 1.5 at baseline (*N* = 52) to 4.0 ± 2.2 at week 12 (*N* = 41) and 3.8 ± 2.2 at last observation carried forward (*N* = 51), with significant changes already by week 4 (−1.5 ± 2.3; 95% CI: −2.1 to −0.8; *p* < 0.001) and sustained at week 12 (last observation carried forward: −1.7; 95% CI: −2.4 to −1.0; *p* < 0.001). Patients with severe pain (NRS ≥ 6) showed more pronounced reductions: −3.0 ± 2.9 (*p* = 0.001) at week 4 and −2.9 ± 2.6 (*p* = 0.003) at week 12. However, among those treated with taxanes (*N* = 12), the reduction was only −0.9 (95% CI: −2.4 to 0.6), which did not reach statistical significance (*p* = 0.204), whereas among those who underwent oxaliplatin treatment (*N* = 29) there was a significant reduction (−1.8; 95% CI: −2.6 to −0.9; *p* < 0.001). This indicates that mirogabalin failed to demonstrate analgesic efficacy specifically for the taxane-exposed subgroup in this exploratory trial. Complementarily, significant improvement in tingling was also observed, with a mean change of −1.2 (95% CI: −1.9 to −0.4, *p* = 0.003) at week 12. Therefore, it appears that mirogabalin can be considered effective in reducing oxaliplatin-induced neuropathic pain and tingling, favoring the continuation of chemotherapy, but the same does not seem to apply to taxanes, demonstrating the complexity of effectively targeting medication treatment for neuropathic pain triggered by different chemotherapeutic agents. However, study limitations such as its exploratory nature, absence of a control group, and small sample size, particularly concerning the percentage of breast cancer survivors and survivors undergoing taxane chemotherapy, indicate the difficulty in generalizing the results.

Although ASCO recommends the use of duloxetine for the management of taxane-induced peripheral neuropathy, the direct extension of this recommendation to TINP still lacks support from more methodologically rigorous studies. Moreover, when tested in clinical trials, duloxetine, mirogabalin, and ALA and ipidacrine showed promising but still insufficient results. These studies presented significant limitations, such as TINP being analyzed as a secondary outcome, small sample sizes, absence of a control group, a low percentage of breast cancer survivors among participants, and high methodological heterogeneity. These limitations hinder the generalization of the findings and the guidance for clinical use of these medications in survivors with TINP.

### 4.2 Neuromodulation

Our search reported four studies published within the last 10 years that used different strategies of neurostimulation to manage TINP, presenting varying effects depending on the specific technique. In an experimental study, Zhang, Gan (22) investigated whether vagus nerve stimulation (VNS) could reduce paclitaxel-induced pain hypersensitivity in a Sprague-Dawley rat model and examine its effects on inflammatory cytokine levels in the DRG. VNS, compared with sham, reduced mechanical hypersensitivity, as assessed by von Frey withdrawal frequency, only 1 day after surgery (*p* = 0.003), but this effect was not maintained for 7 days (*p* = 0.69). Similarly, withdrawal latency in the heat test also increased 1 day after VNS (*p* < 0.001) but returned to the sham group level after 7 days (*p* = 0.80). In the cytokine analysis in the DRG, VNS (vs. sham) significantly increased IL-10 levels 1 day after surgery, with a 5-fold increase (*p* = 0.008), but did not alter TNF-α or NF-*κ*B levels. This increase in IL-10 was also transient, returning to baseline levels after 7 days (*p* = 0.843). It suggests that VNS may transiently attenuate paclitaxel-induced hyperalgesia in animal models through anti-inflammatory mechanisms, possibly related to increased IL-10. Given the short-lived effects on behavior and IL-10 modulation, sustained clinical benefits may require repeated or continuous stimulation, and direct translation to humans should be approached cautiously.

Bakare et al. (2024) conducted a RCT to evaluate the efficacy of electrotherapy with high-tone external muscle stimulation (HTEMS) and transcutaneous electrical nerve stimulation TENS in 51 patients who had previously completed chemotherapy (taxanes or platinum salts) and had established platinum- or taxane-induced peripheral neuropathy (grade ≥ 1). Patients underwent home-based electrotherapy sessions over an eight-week period. The results showed that both TENS and HTEMS significantly improved the sensory (HTEMS: *p* < 0.001; TENS: *p* = 0.006) and motor domains (HTEMS: *p* = 0.015; TENS: *p* = 0.012) of the European Organization for Research and Treatment of Cancer Quality of Life Questionnaire–Chemotherapy-Induced Peripheral Neuropathy 20-Item Scale (EORTC-QLQ-CIPN20). A significant difference was found between HTEMS and the control group for the sensory domain (*p* = 0.039). On the EORTC-QLQ-C30, HTEMS increased physical functioning (*p* = 0.018). Specifically regarding CTCAE, the TENS group improved from grade 3 to 1 (*p* = 0.004) and the HTEMS group improved from grade 2 to 1 (*p* = 0.012). Furthermore, HTEMS increased physical functioning from T0 to T1 (*p* = 0.018), although this difference was not significant when compared to the control group. Therefore, it appears electrotherapy, especially HTEMS, can mitigate the impairment of sensory functions, which may be in line with the improvement in clinical grade. Despite the lack of specific results for TINP, the improvements observed in sensory and motor domains may reflect a beneficial effect on TINP, considering its primarily sensory profile. Methodologically, this study is strengthened by its RCT design, including a control group and a comparison of two types of electrical stimulation. However, the limited sample size may restrict the generalizability of the findings. Future studies employing objective measures and larger cohorts may strengthen and validate these findings, including specific analysis of TINP outcomes.

Sivanesan, Stephens (108) conducted an experimental study in male rats (*N* = 34 enrolled; final *N* = 30) that aimed to examine the inhibitory effect of spinal cord stimulation (SCS) on the development of TINP and gene expression changes in the spinal cord. Behavioral results showed that SCS + paclitaxel (vs. paclitaxel only) significantly reduced taxane-induced mechanical hypersensitivity at multiple time points (day 11 [*p* < 0.001], day 13 [*p* < 0.001], day 15 [*p* = 0.002], day 20 [*p* = 0.03], and day 25 [*p* = 0.01]). However, although SCS partially prevented the decrease in paw withdrawal threshold (PWT) between days 13–25 (*p* = 0.004), it did not completely block the taxane-induced hypersensitivity (day 11 [*p* = 0.009]; day15 [*p* = 0.02]; day 20 [*p* = 0.005]; day 25 [*p* = 0.004]). Interestingly, gene expression analysis in the spinal cord revealed that the SCS + paclitaxel group (vs. Sham SCS + paclitaxel) presented 1,066 differentially expressed genes (7.4%), with 78.4% (836) up-regulated and 21.6% (230) down-regulated. Notably, all 11 genes related to synaptic plasticity were downregulated, which was confirmed by qPCR. In view of this, it appears that SCS inhibits synaptic plasticity by regulating gene networks in the spinal cord, contributing to the mitigation of TINP. This study demonstrated a more sustained therapeutic effect of SCS compared to VNS in the Zhang, Gan (22) study, and for integrating behavioral analysis with large-scale gene expression data (1,066 genes), suggesting deep molecular mechanisms. One limitation, however, is the exclusive use of male rats and the difficulty of translating their findings to humans. Similar analyses should be conducted in translational studies involving human subjects to confirm the clinical relevance of these findings.

Legakis, Bigbee (24) tested the hypothesis that paclitaxel regimens inducing mechanical hypersensitivity in rats would also suppress intracranial self-stimulation (ICSS)-elicited operant responding, a sensitive indicator of reward-related behavior and general well-being. Mechanical sensitivity (von Frey) and performance on the ICSS task were assessed. Consistent across males and females and dose- and time-dependent manner, paclitaxel induced significant mechanical hypersensitivity [day 22 [treated with 0.67 mg/Kg/day], days 8, 22 and 29 [treated with 2.0 mg/Kg/day], days 8, 15, 22, and 29 [treated with 6.0 mg/Kg/day]]; *p* < 0.001). However, crucially, paclitaxel treatment did not alter ICSS responding over time and between groups in most animals (*p* > 0.05). In a follow-up study in 18 male rats with significant mechanical hypersensitivity (*p* < 0.001), ICSS performance was also unchanged (*p* > 0.05). Individual analysis showed that although 6 of 24 treated rats (1 female, 5 male) showed some ICSS suppression, there was no significant correlation between mechanical hypersensitivity and ICSS performance (*r* = 0.105; *p* > 0.05). So, it seems that paclitaxel-induced hypersensitivity in rodents may not be of a type or intensity sufficient to depress ICSS, raising the question of whether withdrawal hypersensitivity observed in animal models is always an accurate assessment of clinical pain. Interestingly, the study appears to demonstrate a clear dissociation between mechanical hypersensitivity and ICSS behavior, suggesting that mechanical hypersensitivity, a common measure in animal models of pain, may not reflect the affective or hedonic aspects of pain that impact ICSS. This highlights the necessity for multimodal approaches that target the different aspects of TINP comprehensively, avoiding reductionist and purely mechanistic interventions.

Invasive procedures for neuropathic pain, especially those involving implantable devices like spinal cord stimulators, carry risks such as infection, hematoma, nerve damage, scarring, and increased pain over time (109). Therefore, there is a pressing need for non-invasive, clinically effective, low-cost, and easily applicable alternatives. In this context, transcranial direct current stimulation (tDCS) and repetitive transcranial magnetic stimulation (rTMS) emerge as promising techniques. tDCS applies a low-intensity direct current (1–2 mA) for approximately 20 min, modulating cortical excitability via anodal (excitatory) or cathodal (inhibitory) stimulation, typically targeting the primary motor cortex (M1) or the dorsolateral prefrontal cortex (DLPFC) (110). rTMS applied over M1 influences a broader pain-related network, including the contralateral M1, thalamus, anterior cingulate cortex, somatosensory cortex, insula, and cerebellum (111). In a recent experimental study involving post-mastectomy breast cancer patients, bilateral tDCS over M1 led to a 32% reduction in pain severity and a 4.8% improvement in shoulder range of motion (112).

Despite these promising findings, a recent systematic review concluded that evidence for tDCS efficacy in cancer-related pain remains insufficient (113). However, a broader scoping review found that tDCS reduced perceived pain in five out of six studies involving cancer patients, with the sole negative result corresponding to a single-session intervention (114). Furthermore, a systematic review reported that combined tDCS and rTMS stimulation of the motor cortex significantly increased pain thresholds in both healthy individuals (ES: 0.16; 95% CI: 0.02 to 0.31; I² = 22.2%) and chronic pain populations (ES: 0.48; 95% CI: 0.15 to 0.89; I² = 68.8%) compared to sham, although effects were not significant when interventions were analyzed separately. Conditioned pain modulation was also significantly improved by the combined protocol (ES: 0.39; 95% CI: −0.64 to −0.14; I² = 17%), but statistical significance was retained only for tDCS when assessed independently (ES: 0.50; 95% CI: −0.85 to −0.15) (115). These results highlight the therapeutic potential of tDCS and rTMS and underscore the urgent need for well-designed RCTs to establish their efficacy in neuropathic pain treatment.

### 4.3 Physical activity and exercise

The American College of Sports Medicine's latest guideline for cancer survivors cites insufficient evidence on physical activity and exercise for pain management, likely due to pain often being treated as a secondary outcome in most clinical trials (116). However, the existing literature indicates that different types of physical activity appear to positively influence central inhibitory pain pathways and the immune system at local, systemic, and neural levels (117). According to Kami, Tajima (118) exercise modulates brain function by activating reward circuits, stimulating glutamatergic neurons in the basomedial amygdala that project to the nucleus accumbens, and suppressing the activation of GABAergic neurons in the central nucleus of the amygdala. These effects collectively reduce negative emotions such as fear and anxiety, thereby contributing to analgesia.

The hypoalgesic responses to different types of exercise (i.e., aerobic and resistance) are attributed to the activity of the endogenous opioid system, the endocannabinoid system, and the interaction between the opioid and serotonergic systems; and appear to be more pronounced in individuals without chronic pain (119). However, there is evidence suggesting that physical exercise is generally beneficial in reducing neuropathic pain intensity. In a systematic review and expert consensus, Zhang, Hu (120) does recommend resistance and balance training as treatment strategies along with the use of exergaming as an adjuvant therapy (Level of evidence II, B) for chemotherapy-induced peripheral neuropathy. Finally, growing evidence suggests that physical activity and exercise may serve as effective strategies for the prevention and treatment of TINP, due to their neuroprotective, anti-inflammatory, and nerve-regenerative effects, demonstrated in both animal models and human studies (121). For example, aerobic exercise improves the oxidative system, by increasing the number of critical antioxidant enzymes (e.g., superoxide dismutase, catalase and glutathione peroxidase) (122) and strengthens the immune system in cancer survivors by enhancing Natural Killer cell cytotoxic activity, lymphocyte proliferation and post-exercise granulocyte count (123). Below, we describe recent clinical trials aimed at improving the understanding of the role of physical activity and exercise in modulating sensory outcomes associated with TINP.

Kleckner, Kamen (124) conducted a secondary analysis of a clinical trial involving 355 participants (79% with breast cancer) who participated in a six-week intervention consisting of daily aerobic and progressive resistance exercise during chemotherapy. The authors observed a group difference favoring the exercise intervention in sensations characteristic of TINP such as thermal sensibility in hands and feet, numbness and tingling. There was less heat/cold sensibility in the hands and feet in the exercise group, with a change score of 0.38 [95% CI: 0.06 to 0.70; *p* = 0.022], compared to 0.77 [95% CI: 0.42 to 1.13; *p* < 0.0001] for the control group. Regarding numbness and tingling, there was a trend toward symptom reduction in the exercise group [change score: exercise = 0.38 [95% CI: 0.04 to 0.71; *p* = 0.027]; control = 0.58 [95% CI: 0.20 to 0.95; *p* = 0.003]]; however, the main effect of exercise did not reach statistical significance (- 0.42, 95% CI: - 0.85 to 0.02; *p* = 0.061). These results suggest that, while physical exercise may offer benefits for sensory symptoms, its effects appear only marginally superior to usual care. However, despite being a RCT, the findings may have been influenced by several limitations, including a small sample size, a relatively short intervention period, and the use of a secondary analysis with subjective symptom assessment based on a simple 0–10 numeric scale, which limits the generalizability of the results.

Bland, Kirkham (125) conducted a RCT with 27 female breast cancer survivors (50 ± 10.2 years; stage I-III or unknown) who received taxane chemotherapy. The intervention consisted of a supervised multimodal exercise program and home-based aerobic exercise, with a total duration of 10 weeks. The comparison between immediate-start (IE) and delayed-start (DE, after chemotherapy) exercise showed that the immediate-start exercise group had significantly less moderate to severe numbness in the toes or feet before cycle 4 (IE: *n* = 1, 9%; DE: *n* = 7, 50%; *p* = 0.04), but not at the end of chemotherapy (IE: *n* = 5, 42%; DE: *n* = 8, 57%; *p* = 1.0). Similarly, vibration sensation was significantly better in the IE group before cycle 4 (*p* = 0.01), but not at the end of chemotherapy (*p* = 0.71) or at follow-up (*p* = 0.13). Overall quality of life (QoL) was significantly better in the IE group at the end of chemotherapy (*p* = 0.05), with a significant group-by-time interaction (*p* = 0.01), although no difference was observed at follow-up (*p* = 0.29). There were no significant between-group differences in other QoL functional subscales such as physical, emotional, or cognitive function. There was a statistical trend toward smaller dose reductions in the IE group (*p* = 0.06), and significantly more patients in the IE group reached the ≥ 85% threshold for relative dose intensity (RDI) (*p* < 0.005), suggesting that exercise may support the maintenance of the prescribed chemotherapy schedule. In both groups, sensory and motor symptoms increased significantly over time (*P* < 0.01). The results indicate a potential benefit of exercise in mitigating some sensory symptoms (e.g., numbness and vibration) at certain times during treatment, but not on the direct impact of the intervention on the intensity or prevalence of TINP *per se*. The relatively small sample size and the absence of an absence of a true non-exercise control group receiving only usual care throughout the entire study duration, compromise the generalization of the results and make it difficult to identify the real effects related exclusively to the exercises. On the other hand, the RCT design comparing different times of intervention initiation increases methodological robustness.

The RCT by Vollmers, Mundhenke (126) compared exercise with usual care in 36 women with breast cancer [[IG] 48.56 ± 11.94 years; [CG] 52.39 ± 10.14 years; stages I-III] receiving primary chemotherapy treatment with paclitaxel. The intervention consisted of regular physical training and sensorimotor exercises during treatment and for six weeks post-chemotherapy. The results showed that the intervention group presented a significant reduction in the sway area during monopodal stance in intervention group at moments T1 and T2 (left leg: *p* < 0.001 [T1], *p* = 0.003 [T2]; right leg: *p* < 0.001 [T1], *p* < 0.001 [T2]). There was also a significant improvement in bipedal postural stability (T1 vs. T0, *p* = 0.039) and in the Fullerton Advanced Balance Scale scores (*p* = 0.004). The intervention prevented the loss of upper limb strength observed in the control group (−1.60 vs. +0.60 Nm; *p* = 0.029). However, no significant differences were observed in lower limb strength (*p* = 0.48), quality of life (*p* > 0.05), neuropathic symptoms (*p* > 0.05) or cancer-related fatigue (*p* > 0.05) between groups. These results indicate that, although regular physical activity combined with sensorimotor exercises improved upper limb stability and strength, this did not translate into a significant impact on general neuropathic symptoms or neuropathic pain. Thus, the integrated therapeutic strategies model for addressing the complex nature of TINP appears to be the most appropriate. However, the small sample size and the fact that neuropathic symptoms were incorporated as secondary outcomes diminish the strength of these results' applicability to TINP.

Andersen Hammond, Pitz (127) investigated the effects of a home-based exercise intervention during chemotherapy on neuropathic pain in 48 breast cancer survivors (stages I-III). A home-based intervention was conducted for 8.25 months (6.6–9.4) with neural gliding exercises, education for management of neuropathic symptoms, stretching/range of motion exercises, and a follow-up call. The results demonstrated that although the proportion of pain-free patients did not reach statistical significance (intervention = 70.1% vs. control = 51%; *p* = 0.053), a reduction was observed in pain over time (Numeric Pain Rating Scale, NPRS) in the intervention group, with a change score of 2 immediately post-chemotherapy and three months post-chemotherapy [OR: 0.85 (95% CI: 0.76 to 0.94; *p* = 0.002)]. Furthermore, although not significant, the painful pressure threshold tended to be higher in the intervention group (intervention = 923 kPa ± 383 kPa vs. control = 744 kPa ± 285 kPa; *p* = 0.034). In a non-randomized subgroup analysis, more physically active participants also exhibited significantly lower heat pain thresholds (left hand: 41.8 [3.08]°C vs. 44.1 [3.47]°C, *p* = 0.021; right hand: 42.5 [2.60]°C vs. 44.4 [3.20]°C, *p* = 0.039) and reduced vibration perception thresholds (left hand: 0.08 [0.04–0.14] µm vs. 0.23 [0.13–0.42] µm, *p* = 0.001; right hand: 0.14 [0.04–0.23] µm vs. 0.24 [0.14–0.49] µm, *p* = 0.001) compared to their less active counterparts. Although it does not appear to be able to eliminate the presence of TINP, the home-based intervention appears to offer relevant clinical benefits in reducing symptoms related to it. The effects appear to be dependent on engagement, with sensory benefits being more prominent in more physically active participants. The sample size was relatively small, given the investigation of multiple outcomes and the exploratory subgroup analysis. On the other hand, the RCT design with evaluation of multiple objectively measured outcomes (dynamometry, algometry, TSAII) increases the methodological quality of the study.

### 4.4 Manual therapy

Generally, massage helps reduce pain by increasing blood flow, promoting muscle relaxation, and releasing analgesic hormones such as serotonin and endorphins, while also decreasing neural excitability. It also lowers cortisol levels and psychological stress, enhancing its analgesic effect and promoting overall well-being (128). In breast cancer survivors at stages I and II, Hernandez-Reif, Ironson (129) demonstrated that a single 30 min massage session led to acute and significant reductions in anxiety (−27%, *p* < 0.01), depression (−75%, *p* < 0.001), and anger (−80%, *p* < 0.01). However, following five sessions distributed over 15 weeks, only anxiety remained significantly reduced (−29%, *p* < 0.01), indicating a more persistent effect on anxious symptoms than on depression or anger. Biochemically, urinary dopamine concentrations increased by 26% [from 258 [110] to 325 [80], *p* < 0.05], and serotonin levels rose by 60% [from 2,114 [1,369] to 3,391 [2,064], *p* < 0.05], suggesting a neurochemical mediation of emotional improvements. Regarding immune function, there was a 12% increase in natural killer (NK) cell counts [from 235 [129] to 263 [95], *p* < 0.05] and a 10% rise in total lymphocyte counts [from 29 [4] to 32 [7], *p* < 0.05], indicating a potential enhancement of immune surveillance.

To date, only one RCT has examined the effects of manual therapy on TINP in breast cancer patients (130). This trial enrolled 40 women with stage II-III breast cancer undergoing adjuvant chemotherapy with paclitaxel, comparing standard care with classical massage (30 min per week: 20 min feet, 10 min hands) administered prior to infusion over 12 weeks. A significant time effect was observed for neuropathic pain in both groups (*p* < 0.001), but with meaningful between-group differences: neuropathic pain prevalence in the control group increased from 0.0% to 57.1% by week 12 (*p* < 0.006), while remaining stable in the massage group (≈10.5%, *p* = 0.955). At week 12, Self-completed Leeds Assessment of Neuropathic Symptoms and Signs (S-LANSS) scores were significantly lower in the massage group (5.68 ± 6.41) compared to the control group (10.83 ± 7.51), with a significant time   ×   group interaction (*p* = 0.041), indicating a more favorable evolution in the massage group.

Additionally, chemotherapy-induced peripheral neuropathy symptoms, assessed via the EORTC QLQ-CIPN20, showed significant increases over time in the sensory (*p* < 0.001) and motor (*p* = 0.002) subscales. However, symptom progression was attenuated in the massage group, with significant between-group differences in sensory (*p* = 0.037), motor (*p* = 0.010), and autonomic (*p* = 0.006) domains, and significant time   ×   group interactions for sensory (*p* = 0.001) and motor symptoms (*p* = 0.004). Mean sensory scores increased moderately in the massage group (27.19 ± 3.76 to 30.99 ± 6.88), but more markedly in the control group (26.45 ± 4.35 to 41.97 ± 18.34). Similarly, motor scores rose slightly in the massage group (26.69 ± 2.48 to 28.19 ± 3.34), indicative of a positive response to massage, but substantially in the control group (26.53 ± 2.89 to 38.48 ± 18.14) (130).

In this study, the application of classical massage during paclitaxel treatment showed strong potential in preventing and modulating TINP, both in terms of its prevalence and symptom severity. Therefore, its integration with other therapeutic strategies appears to be a recommended approach to address the multifaceted nature of TINP symptoms. Despite the small sample size and the challenges in interpreting the nerve conduction studies, the results support classical massage as a non-pharmacological strategy for TINP and CIPN symptoms. The RCT demonstrated strong methodological rigor by applying massage before each chemotherapy infusion and including outcomes such as S-LANSS, EORTC-QLQ-CIPN20, and nerve conduction studies.

### 4.5 Cryotherapy

Studies suggest that cryotherapy, defined by Han, Waddington (46) as the superficial application of cold as a therapeutic agent, is widely used in pain management due to its ability to modulate peripheral neurophysiological processes. Specifically, cryotherapy promotes pain relief by reducing inflammation and edema, oxidative stress, and nerve conduction velocity in pain pathways (131). For example, cryotherapy has been shown to reduce nerve conduction velocity in pain pathways and increased both pain threshold and pain tolerance in the ankles of male athletes (132). In the oncological context, a systematic review showed that cryotherapy is effective in reducing taxane-induced peripheral neuropathy and relieving symptoms such as numbness, tingling, cold sensitivity, and pain; with a recommended protocol involving localized cooling with frozen gloves and socks or continuous-flow systems, starting 15 min before and ending 15 min after chemotherapy (133). In this sense, cryotherapy appears to be most frequently used as a therapeutic strategy for the management of TAPS in cancer patients. Accordingly, in the present study, we conducted a search that identified two studies that investigated the effects of cryotherapy on indirect indicators of TINP, such as grade ≥ 2 peripheral neuropathy, discomfort or therapy discontinuation, and sensory and motor neurotoxicity.

Kanbayashi, Sakaguchi (23) conducted a self-controlled comparative clinical trial with 38 breast cancer survivors (mean = 57.6 ± 11.0) receiving nab-paclitaxel. The intervention consisted of applying cryotherapy to one hand for 60 min (15 min pre to 15 min post infusion) (FG) and compression therapy to the other hand for 90 min (30 min pre to 30 min post infusion) using two tight surgical gloves (SG) during chemotherapy. The results indicated that both FG and SG appeared effective in preventing nab-paclitaxel-induced peripheral neuropathy and demonstrated comparable results. The frequency of CTCAE grade ≥ 2 sensory and motor neurotoxicity was 18% (*n* = 7) in both FG and SG groups. For PNQ grade ≥ D, the frequency was 2.63% (*n* = 1) in both groups (sensory and motor domains). There were no statistically significant differences between FG and SG in Patient Neurotoxicity Questionnaire (PNQ) sensory scores (*p* = 0.32–1.0), PNQ motor scores (*p* = 0.51–1.0) or Functional Assessment of Cancer Therapy-taxane total scores (*p* = 0.67–0.93). Fingertip temperature decreased significantly in both groups compared to baseline (FG: 8.3–9.8°C, SG: 3.0–3.8°C, both *p* = 0.0001), with post-treatment temperature in the FG hand being significantly lower (22.3°C) than in the SG hand (27.9°C, *p* = 0.0001). Although the findings address sensorimotor aspects, the study addressed general neuropathy and not TINP directly, which makes it difficult to extrapolate the results in this regard. In addition, the lack of a control group and the small sample size also constitute important limitations. On the other hand, the self-controlled design potentially reduces variability, and objective fingertip temperature measurements provide greater reliability to the results.

In another study, Brunner, Emmelheinz (134) conducted a prospective randomized trial with the primary objective of demonstrating the superiority of cryocompression (CC) over cryotherapy alone (C) in preventing taxane-induced peripheral neuropathy (grade ≥ 2) in cancer survivors (*n* = 194; 77.3% with breast cancer). The intervention (CC or C, using Hiotherm cooling devices and surgical gloves for CC) was initiated 30 min before, continued throughout the infusion, and for an additional 30 min after completion. At baseline, 95% of patients in both groups had grade 0 peripheral neuropathy. The results showed no statistically significant difference between the CC and C groups regarding the prevalence of grade ≥ 2 taxane-induced peripheral neuropathy over time up to 6–9 months post-chemotherapy (Chi-square test *p*-values: T1 = 0.313; T2 = 0.727; T3 = 0.589; T4 = 0.295). Similarly, no significant differences were observed between the groups in neurological assessments or in either of the quality-of-life questionnaires (for all, *p* > 0.1). Although both interventions significantly reduced skin temperature, cryocompression was not superior in preventing CIPN. Notably, the CC group exhibited a significantly higher rate of therapy discontinuation due to discomfort compared to the C group (23.7% vs. 11.3%, *p* = 0.0234). The authors suggest that both cryotherapy and cryocompression are safe and may reduce CIPN relative to historical data. However, cryocompression was not more effective and demonstrated lower tolerability. The randomized design, the considerable sample size and the assessment of multiple CIPN outcomes provide methodological robustness to the study. However, comparisons only between C and CC suggest the need for a future RCT that includes a control group of usual care to provide greater capacity for extrapolation of the results. In addition, failure to demonstrate the superiority of cryocompression as the primary endpoint and the high discontinuation rate in the CC group, likely introduce bias, and are important limitation.

### 4.6 Acupuncture

Acupuncture modulates the somatosensory system to relieve neuropathic pain by targeting ion channels (e.g., TRPV1, P2X3, NaV1.3, NaV1.7, and NaV1.8) and pain-associated receptors (e.g., CX3CL1), thereby reducing neuronal sensitization and inflammation (135). This mechanism also inhibits the activation of glial cells in the spinal cord, contributing to the disruption of processes that sustain chronic pain (Ma, Chen, et al. 2022). In a preclinical study, Li, Yin (136) demonstrated that electroacupuncture significantly attenuates mechanical and thermal hyperalgesia associated with taxane administration. From a biochemical and immunological standpoint, electroacupuncture has been shown to modulate key molecular targets implicated in TINP, including the downregulation of TLR4 and MyD88, suppression of TRPV1 expression and activity, and inhibition of astrocyte and microglial activation in the spinal cord (136).

From a clinical perspective, only two clinical studies evaluating acupuncture for the management of TINP in breast cancer survivors were identified. The phase IIA single-arm trial by Bao, Seidman (137) evaluated weekly acupuncture during the chemotherapy period in 27 patients with breast cancer (stages I-III) who developed grade II taxane-induced peripheral neuropathy. Results showed that 33% remained at Grade II, 4% progressed to Grade III, while 41% and 22% improved to Grades I and 0, respectively. Despite the low progression to Grade III, neuropathic symptoms worsened during the screening phase: mean neuropathic pain scores (NPS) increased from approximately 9 (95%CI: 8 to 10.5) at baseline to approximately 17 (95%CI: 15.5 to 18.5) and FACT-GOG-Ntx scores declined from approximately 39 (95% CI: 38 to 40) to approximately 33 (95% CI: 31.5 to 34.5). However, during the acupuncture intervention, both NPS and FACT-GOG-Ntx scores stabilized and remained unchanged at 3-month follow-up, suggesting a potential benefit in symptom control. Vibration sense tests (mean difference = 0.49; 95%CI: −1.61 to 2.60; *p* = 0.06) and BDNF levels (mean difference = 148; 95%CI: −740 to 1,035; *p* = 0.7) showed no significant changes pre- and post-intervention. These findings suggest that acupuncture may offer relevant symptomatic stabilization for patients with TINP, even in the absence of objective neurological reversal. This strengthens its role as a complementary therapeutic approach within an integrative management model, particularly when conventional therapies are poorly tolerated. The study provides important preliminary data on the use of acupuncture in TAPS, with comprehensive methodological descriptions including specific application techniques and multiple outcome measures. However, the lack of a control group limits the causal attribution between the intervention and the improvement in neuropathic symptoms, reinforcing the need for RCTs to validate its efficacy. Furthermore, interventions applied at later stages following chemotherapy should be investigated to better determine the true efficacy of acupuncture in the management of taxane-induced peripheral neuropathy (TINP).

In the three-arm RCT by Zhi, Baser (138) 63 patients (56% breast cancer in stage I-IV) received taxane-based chemotherapy (54%) or taxane and platinum-based chemotherapy (21%), and the remaining portion received other protocols included in the trial's scope. The interventions were composed of (1) real acupuncture (RA), (2) sham acupuncture (SA), or (3) usual care (UC) over eight weeks. The neuropathic pain assessments at baseline (NRS) were 4.3 (RA), 4.0 (SA), and 4.6 (UC). While changes in NRS pain scores were not reported, psychophysical quantitative sensory testing (QST) showed significant benefits of RA and SA over UC in preserving vibration detection in the feet at week 8 [RA-UC: −6.88 [95%CI: −12.59 to −1.17], *p* < 0.05; SA-UC: −5.61 [95%CI: −11.13, to 0.10], *p* < 0.05], and in cold detection thresholds [RA-UC: 2.14 [95%CI: 0.57 to 3.71], *p* < 0.01; SA-UC: 1.95 [95%CI: 0.42 to 3.48], *p* < 0.05]. For cold pain thresholds, RA showed less deterioration than UC at week 12 [RA-UC: −4.41 (95%CI: −8.16 to −0.66), *p* < 0.05]. Other QST measures revealed no significant differences. The results indicate that both real and sham acupuncture provide sensory benefits compared to usual care, with real acupuncture being superior in preventing cold-induced pain exacerbation. This reinforces the idea that acupuncture interventions may have clinical relevance in the management of TINP. Interestingly, the lack of significant improvement in self-reported pain (SRP) may reflect a latency in subjective pain perception or a dissociation between neuronal damage and immediate pain symptoms. The three-arm RCT design of this study allowed for rigorous assessment of the effect of acupuncture, placebo effect, or patient expectations. Furthermore, the use of semi-objective quantitative sensory testing (QST) and subjective reports (NRS) also added extrapolation power to the results. However, the sample size is relatively small and the lack of significant differences between groups in changes from baseline in most QST measures, except for a few specific measures at specific time points suggests a limited objective impact on broadly assessed sensory functions.

## 5 Discussion

TINP remains a frequent, debilitating, and therapeutically challenging adverse effect in breast cancer survivors, with substantial impacts on physical function and quality of life. Despite its prevalence, pharmacological management remains limited. Duloxetine is the only agent with strong clinical evidence for the treatment of taxane-induced peripheral neuropathy; however, its efficacy for specifically managing TINP remains uncertain. Other pharmacological strategies, such as mirogabalin and the combination of alpha-lipoic acid with ipidacrine, have demonstrated preliminary benefits but require validation through more rigorous trials.

Furthermore, the clinical relevance of TAPS must be integrated into the interpretation of these findings. TAPS is increasingly recognized not merely as a transient side effect, but as a potential clinical predictor or mechanistic precursor to persistent TINP (13, 16, 79). The inherent difficulty in clinically distinguishing TAPS from TINP in several reviewed trials, particularly those assessing cryotherapy or acupuncture, suggests that observed therapeutic benefits may partly stem from the mitigation of the early neurotoxic and inflammatory cascade (23, 134, 137). Consequently, addressing these acute symptoms may be critical in limiting subsequent central sensitization and the transition to chronic pain.

Considering these limitations, there is growing interest in complementary and integrative therapeutic strategies. A mechanistic understanding of the somatosensory system, encompassing both the peripheral and central nervous systems, is essential to inform hypothesis-driven research and guide the design of translational RCTs. While much of the current literature lacks a mechanistic foundation, non-pharmacological interventions have shown promising clinical and physiological outcomes. For instance, physical activity has demonstrated antioxidant, neuroprotective, anti-inflammatory, and analgesic effects, contributing to the preservation of neuromuscular function. Electrical stimulation techniques, such as VNS, SCS, and TENS/HTEMS, have yielded improvements in both sensory symptoms and functional outcomes. Manual therapies, particularly classical massage, have been effective in alleviating TINP and related motor and autonomic symptoms. Other modalities, such as cryotherapy and non-invasive neuromodulation (e.g., tDCS, rTMS), are emerging as promising options but still require high-quality clinical evidence.

However, caution is needed when interpreting the results presented in this review, since the methodological quality of existing studies is highly variable, ranging from preclinical models to small-scale RCTs with heterogeneous designs. This heterogeneity limits statistical power and compromises the generalizability of findings, highlighting the urgent need for well-designed, large-scale RCTs with standardized protocols, adequate control conditions, and clinically meaningful outcomes.

Taken together, current evidence underscores the need for a personalized, multimodal approach to managing TINP. Such an approach should consider individual pain phenotypes, treatment phase, and patient functionality. Integrating multiple patient-centered modalities may improve symptom control, reduce reliance on pharmacotherapy, and enhance overall quality of life. Given the complexity of TINP, interdisciplinary care and the combination of evidence-based interventions represent a promising and necessary path forward. Future research should prioritize translational designs that explore both individual and synergistic effects of multimodal interventions to establish effective, accessible, and sustainable strategies for the long-term management of TINP.
